# Current insights into the expression and functions of tumor-derived immunoglobulins

**DOI:** 10.1038/s41420-021-00550-9

**Published:** 2021-06-28

**Authors:** Jing Zhao, Hui Peng, Jie Gao, Anna Nong, Haoming Hua, Shulin Yang, Liying Chen, Xiangsheng Wu, Hao Zhang, Juping Wang

**Affiliations:** Department of Pathophysiology, School of Basic Medical Sciences, Youjiang Medical University for Nationalities, 533000 Baise, China

**Keywords:** Diagnostic markers, Immunoediting

## Abstract

Numerous studies have reported expressions of immunoglobulins (Igs) in many human tumor tissues and cells. Tumor-derived Igs have displayed multiple significant functions which are different from classical Igs produced by B lymphocytes and plasma cells. This review will concentrate on major progress in expressions, functions, and mechanisms of tumor-derived Igs, similarities and differences between tumor-derived Igs and B-cell-derived Igs. We also discuss the future research directions of tumor-derived Igs, including their structural characteristics, physicochemical properties, mechanisms for rearrangement and expression regulation, signaling pathways involved, and clinical applications.

## Facts

- Classical Igs are produced by B lymphocytes.
- A lot of tumor cells express Igs by themselves.
- Tumor-derived Igs have some specific functions which are different from B-cell-derived Igs, while the mechanisms for the above functions of tumor-derived Igs are unclear.

## Open questions

- Can any tumor express Igs?
- Which cell signaling pathways are involved in tumor-derived Ig-promoted tumor cell growth and proliferation?
- What are the mechanisms by which tumor-derived Igs augment cell migration, invasion, and metastasis?
- What are the mechanisms by which tumor-derived Igs facilitate tumor immune escape?
- Are tumor-derived Igs different from B-cell-derived Igs?
- What are the clinical applications of tumor-derived Igs?

## Introduction

Ig, a class of globulin with antibody activity, is an important component of disease resistance in the body. It is widely found in blood, tissue fluid, and exocrine fluid, accounting for ~20% of the total plasma protein [1–4]. Five classes of Igs, namely IgA, IgG, IgM, IgE, and IgD, are found in the human body. Each class consists of two identical Ig heavy chains and two identical Ig light chains, which are linked by disulfide bridges to form a “Y”-shaped molecule with twofold symmetry. Ig heavy chains are classified into five major isotypes including Igα, Igγ, Igμ, Igε, and Igδ, and each is specific to its Ig class (IgA, IgG, IgM, IgE, and IgD). Among above Ig heavy chains, Igγ heavy chains are split into four subtypes including Igγ1, Igγ2, Igγ3, and Igγ4, so IgG has four subtypes (IgG1, IgG2, IgG3, and IgG4) according to its corresponding heavy chains. Igα heavy chains are similarly split into two subtypes including Igα1 and Igα2, so IgA contains two subtypes (IgA1 and IgA2) according to its corresponding heavy chains. Ig light chains are classified into two isotypes namely Igλ and Igκ, and Igλ is further classified into Igλ1, Igλ2, Igλ3, and Igλ4 [5–9]. According to traditional theory, only B lymphocytes and plasma cells can produce and secrete Igs [10, 11]. However, many research teams recently found that various cancer cells (such as breast cancer, lung cancer, and cervical cancer) can also express Igs, especially IgG [12–15]. At present, more and more studies have shown that tumor-derived Igs play a very important role in the occurrence and development of tumor cells [15–18]. Therefore, further investigation of the roles of tumor-derived Igs in the process of various tumor pathological changes will be helpful to formulate new strategies for the prevention, diagnosis, and treatment of tumors.

### Expression of tumor-derived Igs

#### Expression of tumor-derived Ig heavy chains

The finding of Ig heavy chains in tumor cells dates back to1998. In that year, Kimoto used reverse transcription (RT)- nested PCR to test gene transcripts for the heavy-chain constant regions of IgM, IgD, IgG3, IgG1, IgE, IgA, and T-cell receptor-α in five carcinoma cell lines, including SW1116, HEp2, MCF-7, MDA-MB-231, and HC48 [19]. Several years later, other research groups also confirmed the expressions of heavy chains in many tumor tissues and cell lines. A few human Ig heavy-chain constant regions were detected in human hepatocellular carcinomas using cDNA microarray, including Ig heavy constant gamma 3 (IGHG3), Ig heavy constant alpha1 (IGHA1), Ig heavy constant mu (IGHM) [20]. Ig heavy-chain V–II region was detected in protein level using two-dimensional electrophoresis in human nasopharyngeal carcinoma cell lines [21]. it was reported that IgA heavy-chain expression was examined using immunohistochemistry (IHC), western blot (WB), and enzyme-linked immunosorbent assay (ELISA) in seven human different cancer cell lines, including MCF-7, SW480, MGC, HeLa, HNE2, CNE1-LMP1, and Tet-on-LMP1-HNE2. The results showed that IgA heavy-chain protein was expressed in the above cancer cells and their supernatants [22]. Zheng et al. found a colorectal cancer-associated gene SNC73 which encoded a peptide identical to the constant region of an IgA molecule [23, 24]. They later found expression of SNC73 in gastric cancer, breast cancer, lung cancer, liver cancer, and noncancerous tissues, but the difference of SNC73 expression in the above cancerous tissues and noncancerous tissues was not significant [25]. In addition, they found that the human colon cancer cells (SW480) expressed SNC73, Ig heavy chain α1, recombination activating gene 1 (RAG1), and RAG2 [26]. IgG was reported to express in a number of epithelial malignant tumor cells, such as human breast cancer, colorectal cancer, and liver cancer [16]. In that study, researchers found that IgG was not only localized in the cytoplasm or on the plasma membrane of these cells but also was secreted into cell culture medium [16]. Ig heavy-chain variable region gene expression was examined using nested reverse transcription-PCR in six breast cancer cell lines (BT474, MDA-MB-231, MCF-7, SKBR3, T47D, and ZR75-1) [27]. The above gene transcripts were identifiable in four of six cell lines. IgG heavy chains including variable regions were reported to be expressed in several kinds of carcinoma cell lines, including HT-29, A549, BCL-7402, HeLa S3, and PC3, in both protein and mRNA levels by IHC, in situ hybridization (ISH), and laser microdissection-assisted nested RT-PCR [14, 28]. Zheng et al. demonstrated that human epithelial cancer cells, including HeLa, CNE1, MGC, MCF-7, and SW480, expressed Igα heavy chain. They found that the above cancer cells had the recombination VDJ region and the essential effectors including RAG1 and RAG2, except for activation-induced cytidine deaminase (AID) protein. Moreover, they confirmed that the above cancer cells not only expressed the protein of Igα heavy chain but also secreted the protein into cell culture media [29].

**Fig. 1:**
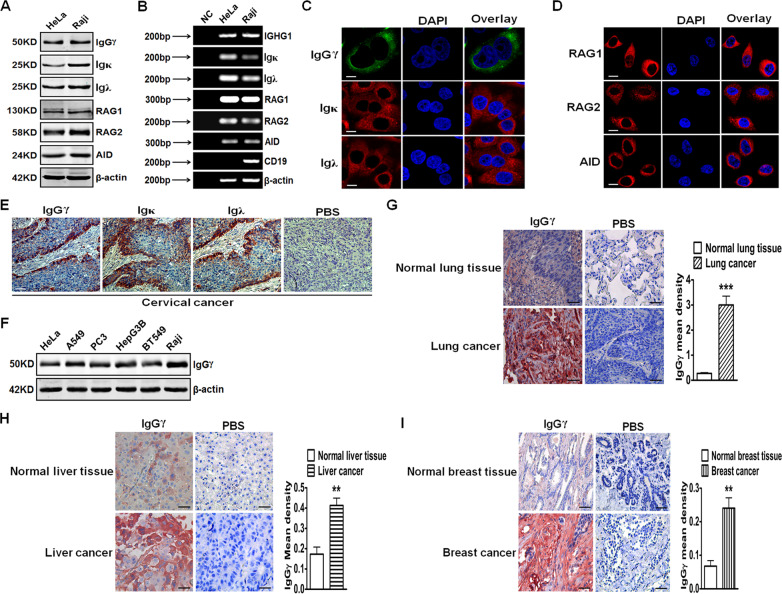
Expression and distribution of IgG in multiple cancer cells and tissues. **A** Expressions of IgG Heavy chain (γ), light chains (λ, κ), and other related proteins such as RAG1, RAG2, AID were tested with WB in HeLa cells. Raji cells were used as a positive control. β-actin was used as an internal control. **B** The above molecule transcripts were measured with RT-PCR. DNase-treated RNA without adding reverse transcriptase was used as a negative control (NC). CD19 was a B lymphocyte marker. **C**, **D** Distribution of the above molecules was examined with IF (scale bar, 20 μm). **E** Expressions of IgG heavy chain (γ), light chains (λ, κ) in cervical cancer tissues (scale bar, 50 μm). PBS was used as a negative control. **F** IgG expression at the protein level in HeLa, HEp2, PC3, Hep3B, BT549, and Raji cells. **G**, **H**, **I** The analytic comparison of IgG heavy-chain (γ) expression in normal tissues (lung, liver, breast) and their cancer tissues (scale bar, 20 μm). IgGγ mean densities were analyzed in the right panel. PBS was used as a negative control. Reproduced from Wang et al. [15].

In 2008, IgG and IgA proteins were found in the cytoplasm or plasma membrane or secretion of malignant cells and pleomorphic adenoma tumor cells. In addition, researchers also found rearranged Ig gene transcripts in the above tumor cells [30]. Many tumor cells or tissues were confirmed to express Ig heavy chains by WB, nested RT-PCR, IHC, or ISH, including breast cancer [12, 13, 31, 32], cervical cancer, colon cancer, liver cancer, lymphoma, melanoma, neuroblastoma, soft tissue lesions [33], soft tissue sarcoma [34], acute myeloid leukemia (AML) [35, 36], ovarian cancer, papillary thyroid cancer [37], prostate cancer [38, 39], urothelial carcinoma [40], clear cell renal cell carcinoma (ccRCC) [41], bladder cancer [42], salivary adenoid cystic carcinoma (SACC) [43, 44], lung cancer [45, 46], colorectal cancer [17, 47], colon cancer [17, 48], pancreas cancer [18], pancreatic ductal adenocarcinoma [49, 50], parathyroid cancer [51], and intraductal papillary mucinous neoplasms [52]. Our previous results demonstrated IGHG1 expression in both protein and mRNA levels in cervical cancer tissues and cell lines, including HeLa and SiHa by RT-PCR, WB, IF, and IHC [15] (Fig. 1).

### Expression of tumor-derived Ig light chains

Many studies have shown that light chains, including κ and λ, were expressed in a variety of cancer cells. Li et al. analyzed Igκ constant region mRNA expression in different stages of cervical tissues using ISH. The results showed that the expression of mRNA for the κ constant region in epithelia with dysplasia and carcinoma was higher than that in epithelia with cervicitis. Igκ was localized in both the cytoplasm and nuclei of epithelial cells [53]. Igκ chain expression was confirmed in nasopharyngeal carcinoma cell line HNE2 by WB, RT-PCR, and flow cytometric analysis [54, 55]. Igκ light chain was also reported to be expressed in other tumor cells including breast cancer [13, 14, 31, 56–58], colorectal cancer [16, 17, 26, 32, 47, 56, 58, 59], lung carcinoma [14, 56, 58], AML [36, 60], hepatocellular cancer [14, 32, 58], cervical cancer [14, 16, 31, 32, 53, 58, 59], prostate cancer [14, 31, 38, 59], gastric cancer [61], papillary thyroid cancer [37], soft tissue tumors [32–34], pancreas cancer [18, 49], and nasopharyngeal carcinoma [62, 63].

The expression of the Igλ light chain was also examined. Researchers analyzed the expressions of Igλ and Igκ light chains in gastric cancer cells in 22 human gastric cancer tissue specimens by the IHC method–LSAB method. The results demonstrated only Igλ expression in 1 (4.5%) and co-expression of Igλ and Igκ light chains in 17 (77.3%) among all specimens, suggesting that co-expression of Ig Igλ and Igκ light chains in gastric cancer cells was common [61]. Igλ light chain was also found to be expressed in other tumor cells, including hepatocellular cancer [20], pancreas cancer [18, 49], breast cancer [31], cervical cancer [31, 59], prostate cancer [31, 59], gastric cancer [61], colorectal cancer [59], and AML [36]. The tumor tissues and cell lines that express Ig heavy chains and/or light chains are summarized in Table 1.

**Table 1:** List of tumor tissues and cell lines expressing Ig heavy chains and/or light chains.

Structures of Igs	Cancer tissues or cell lines	References
Ig heavy chains	Breast cancer tissue; BT474, MDA-MB-231, MCF-7, SKBR3, T47D, ZR75-1, MDA-435, MDA-MB-468	[12, 13, 16, 19, 22, 25, 27, 29, 31, 32]
	Cervical cancer tissue; HeLa, HeLa MR, SiHa, C-33A, CA33, ME-180	[15, 22, 29, 31, 32]
	Colorectal cancer tissue; HT-29, SW480, LOVO, HCT116, SW1116, HCT115, SW-48, SW620, HCT-8	[16, 17, 19, 22–24, 26, 28, 29, 31, 32, 47, 48]
	Liver cancer tissue; Hep3B, HepG2, HEp-2	[16, 20, 21, 25, 31, 32]
	Pancreatic cancer tissue; HC48	[18, 19, 32]
	Laryngeal squamous cell carcinoma cell line: HEp2	[19]
	Gastric cancer tissue; MGC	[22, 25, 29]
	Nasopharyngeal cancer tissue; HNE2, CNE1-LMP1, Tet-on-LMP1-HNE2	[22, 29]
	Lung cancer tissue; A549, Calu-6, H441	[25, 31, 32, 45, 46]
	Lymphoma cell line: HEL1	[31]
	Melanoma cell lines: MMAN, MMRU, SK-Mel-3	[31]
	Neuroblastoma cell line: SHSY5Y	[31]
	Ovarian cancer tissue; SK-OV-3, OC-3-VGH	[31, 32]
	Prostate cancer tissue; PC3, Du145, LNCaP	[31, 38, 39]
	Soft tissue tumor tissue; A673, U2OS, HT1080	[31–34]
	Acute myeloid leukemia tissue; HEL, HL-60, KG-1,NB4, THP-1, OCI-AML3	[35, 36]
	Papillary thyroid cancer tissue	[37]
	Urothelial carcinoma tissue; T24, BIU-87	[40]
	Clear cell renal cell carcinoma tissue; 786-0, ACHN, CAKI-I	[41]
	Bladder cancer tissue; 5637, BIU-87, EJ	[42]
	Human salivary adenoid cystic carcinoma tissue; SACC-83	[43, 44]
	Pancreatic cancer tissue; PANC-1, BxPC-3	[49, 50]
	Parathyroid cancer tissue	[51]
	Intraductal papillary mucinous neoplasms tissue	[52]
Ig light chains	Breast cancer tissue; MCF-7, MDA-MB-231, MDA-435, T47D,MDA-MB-468,SW-48	[13, 14, 31, 56–58]
	Cervical cancer tissue; HeLa S3,HeLa MR,C-33A, CA33, ME-180, SiHa	[14–16, 31, 32, 53, 58, 59]
	Hepatocellular cancer tissue; HepG2	[14, 20, 32, 58]
	Prostate cancer tissue; PC3, Du145, LNCaP	[14, 31, 38, 59]
	Lung cancer tissue; A549	[14, 56, 58]
	Colorectal cancer tissue; HT-29, LOVO,SW116,HCT116	[16, 17, 26, 32, 47, 56, 58, 59]
	Pancreatic cancer tissue; MIA PaCa-2, PANC-1, AsPC-1, BxPC-3	[18, 49]
	Soft tissue tumor tissue; U2OS, A673, HT1080	[32–34]
	Papillary thyroid cancer tissue	[37]
	Acute myeloid leukemia tissue; HEL, HL-60, KG-1, NB4, OCI-AML3, THP-1	[36, 60]
	Nasopharyngeal cancer tissue; HNE2	[54, 55, 62, 63]
	Gastric cancer tissue	[61]

### Molecular regulatory mechanisms of tumor-derived Ig expression

B-cell-derived Igs are assembled by V(D)J recombination, which includes randomly choosing a pair of V, D, J gene segments, production of double-strand breaks (DSB) adjacent to each segment, deletion or invertion of the intervening DNA and ligation of the segments together, during lymphocyte development [64]. This recombination is initiated by DSB produced by RAG1 and RAG2 at specific recombination signal sequences [65]. Numerous studies have shown that RAG1 and RAG2 were found to be expressed at both protein and mRNA levels in many cancer cell lines, including colon cancer, lung cancer, cervical cancer, liver cancer, prostate cancer, nasopharyngeal cancer, gastric cancer, breast cancer, papillary thyroid cancer, pancreatic cancer, ovarian cancer, soft tissue sarcoma, and lymphoma [14, 16, 18, 29, 31, 34, 37, 38, 58, 66]. AID, which is required for antibody maturation involving somatic hypermutation, class switch recombination, and Ig gene conversion in some species, is also linked to tumorigenesis. It was reported to express in some cancer cells, including papillary thyroid cancer, pancreatic cancer, ovarian cancer, breast cancer, soft tissue sarcoma, cervical cancer, lymphoma, and colorectal cancer using nested RT-PCR [14, 17, 18, 27, 31, 34, 37, 67]. In addition, I_H_-C_H_ germ-line switch transcripts (Iγ-Cγ, Iα-Cα) were also detected in breast cancer cells (BT474, T47D), soft tissue sarcoma cells (A673, U2OS, and HT1080), pancreatic cancer cells (MIA PaCa-2, PANC-1, AsPC-1, and BxPC-3), cervical cancer cells (HeLa S3), liver cancer cells (Bel-7402), and lymphoma cells (Raji) [14, 18, 27, 34]. The tumor tissues and cell lines that express RAG1, RAG2, AID, and I_H_-C_H_ germ-line switch transcripts are summarized in Table 2.

**Table 2:** List of tumor tissues and cell lines expressing Ig-related genes.

Ig-related genes	Cancer tissues or cell lines	References
RAG1	Breast cancer tissue; MCF-7, BT474 MDA-MB-231, SKBR3, T47D, ZR75-1, Bcap-37	[14, 16, 18, 29, 58, 66]
	Colon cancer cell tissue; HT-29, LOVO, SW116, SW480, HCT116	[14, 16, 17, 29, 58]
	Cervical cancer tissue; HeLa S3, HeLa MR	[16, 29, 58]
	Lung cancer tissue; A549	[16, 66]
	Ovarian cancer tissue; CaOV3, OC-3-VGH	[16, 31]
	B lymphocytic leukemia tissue; Raji, Daudi	[16, 29, 66]
	Esophagus carcinoma cell line: HEp2	[14]
	Nasopharyngeal carcinoma cell line: CNE1	[29]
	Gastric cancer cell line: MGC	[29]
	Thyroid cancer tissue	[36]
	Prostate cancer tissue; PC3, LNCaP, Du145	[37, 66]
	Ewing’s sarcoma cell line: A673	[33]
	Osteosarcoma cell line: U2OS	[33]
	Hepatoma cell line: SMMC-7721	[58]
RAG2	Breast cancer cell tissue; MCF-7, BT474, MDA-MB-231, SKBR3, T47D, ZR75-1, Bcap-37	[14, 16, 18, 29, 58, 66]
	Colon cancer cell tissue; HT-29, LOVO, SW116, SW480, HCT116	[14, 16, 17, 29, 58]
	Cervical cancer tissue; HeLa S3, HeLa MR	[16, 29, 58]
	Lung cancer tissue; A549	[16, 66]
	Ovarian cancer tissue; CaOV3, OC-3-VGH	[16, 31]
	B lymphocytic leukemia tissue; Raji, Daudi	[16, 29, 66]
	Esophagus carcinoma cell line: HEp2	[14]
	Nasopharyngeal carcinoma cell line: CNE1	[29]
	Gastric cancer cell line: MGC	[29]
	Thyroid cancer tissue	[36]
	Prostate cancer tissue; PC3, LNCaP, Du145	[37, 66]
	Ewing’s sarcoma cell line: A673	[33]
	Osteosarcoma cell line: U2OS	[33]
	Hepatoma cell line: SMMC-7721	[58]
AID	Breast cancer cell lines: MCF-7, BT474, SKBR3, T47D, ZR75-1, MDA-MB-231,	[14, 27, 29, 67]
	Lung cancer cell line: A549	[14]
	Cervical cancer cell line: HeLa S3	[14, 29]
	Liver cancer cell line: BCL-7402	[14]
	Prostate cancer cell line: PC3	[14]
	Burkitt lymphoma cell line: Raji	[14, 29]
	Nasopharyngeal carcinoma cell line: CNE1	[29]
	Gastric cancer cell line: MGC	[29]
	Colon cancer cell line: SW480	[29]
	Thyroid cancer tissue	[36]
	Ovarian cancer cell line: OC-3-VGH	[31]
	Ewing’s sarcoma cell line: A673	[33]
	Osteosarcoma cell line: U2OS	[33]
	Colorectal Cancer tissue; LOVO, HCT116	[17]
I_H_-C_H_	Cervical cancer cell line: HeLa	[29]
	Nasopharyngeal carcinoma cell line: CNE1	[29]
	Gastric cancer cell line; MGC	[29]
	Breast cancer cell lines: MCF-7, BT474, MDA-MB-231, SKBR3, T47D, ZR75-1	[18, 29]
	Colon cancer cell line: SW480	[29]
	Burkitt lymphoma cell line: Raji	[29]
	Myeloma cell lines: XG-2, XG-7	[29]

**Fig. 2:**
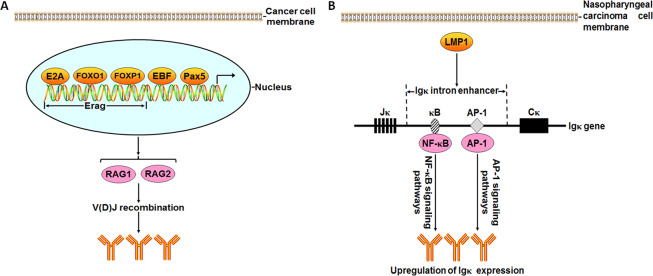
Schematic illustration of the regulatory mechanisms of tumor-derived Ig expression. **A** The transcription factors (E2A, FOXO1, FOXP1, EBF, and Pax5) bond to Erag and other RAG promoters to activate transcription of RAG1 and RAG2, which in turn promoted V(D)J recombination of Igs in cancer cells. Finally, tumor-derived Ig expression was upregulated. **B** LMP1 enhanced Igκ intron enhancer activity, which in turn promoted transcription factors NF-κB (p52 and p65) binding to its corresponding motif as well as AP-1 (c-Jun and c-Fos) binding to its corresponding motif in Igκ gene in NPC cells. Finally, Igκ expression was upregulated through the activation of NF-κB and AP-1 signaling pathways.

The transcription factors play a vital role in the regulation of tumor-derived Ig expression. At present, the transcription factors of the B-cell-derived IgG gene include octamer-related protein 1, 2 (Oct-1, 2), nuclear factor kappa B (NF-κB), B-cell Oct-binding factor-1, etc., while there is no Oct-2 among the transcription factors of tumor-derived IgG. Tumor-derived IgG promoter can be activated, and its two novel positive regulatory elements are located at −800 to −610 bp and at −610 to −300 bp, respectively [68–72]. Geng et al. explored the expression of a transcript of the Igα1 gene SNC73 and its related genes (RAG1, RAG2, and 3 transcription factors) in cancer cells, including LOVO (colorectal cancer), SW480 (colorectal cancer), HeLa (cervical cancer), SMC7721 (liver cancer), and Bcap-37 (breast cancer). The results showed that SNC73, RAG1, and RAG2 were detected in all of the above cancer cells. The above three transcription factors of RAG1 or RAG2 were EBF, E2A, and Pax5, respectively. EBF was detected at the mRNA level in the above cancer cells, and Pax5 was only expressed in SW480 cells. However, none of the above five tumor cell lines expressed E2A [58, 73]. Chen et al. reported that three transcription factors of RAG1 (E2A, FOXO1, and FOXP1) were expressed in cancer cells (A549, PC3, MCF-7, and MDA-MB-231) and located in the nuclei of these cells. Upregulated expressions of E2A, FOXO1, or FOXP1 enhanced RAG1 expression, while silencing of E2A, FOXO1, or FOXP1 decreased RAG1 expression in the cancer cells. Histone H3 acetylation and E2A, FOXO1, FOXP1 binding to RAG enhancer (Erag) were confirmed in MCF-7 using chromatin immunoprecipitation. The above results demonstrated that the transcription factors E2A, FOXO1, and FOXP1 regulated RAG1 expression by binding to Erag, which in turn initiated Ig gene rearrangement in cancer cells [66]. The above regulatory mechanisms of tumor-derived Ig expression are illustrated in Fig. 2A.

The mechanisms of tumor-derived Ig expression mediated by some specific signaling pathways have been reported. Cao et al. found that EBV-encoded latent membrane protein 1 (LMP1) upregulated Igκ expression in nasopharyngeal carcinoma (NPC) cells. The inhibitors of JNK and NF-κB (Bay11-7082 and SP600125) attenuated LMP1-enhanced Igκ expression in NPC cells, respectively. Compared with their parental cells, LMP1-positive NPC cells, which expressed the dominant-negative mutant of IκBα or c-Jun, displayed markedly low levels of Igκ production. These results indicated that LMP1 strengthened Igκ expression through NF-κB and activator protein-1 (AP-1) signaling pathways [54]. In addition, Cao et al demonstrated that Igκ intron enhancer was active in Igκ-expressing NPC cells. LMP1 increased Igκ intron enhancer activity, which was involved in NF-κB and AP-1 signaling pathways. LMP1 accelerated transcription factors p52 and p65 binding to the NF-κB motif as well as c-Jun and c-Fos binding to the AP-1 motif in vitro and in vivo. The above results suggested that LMP1 augmented Igκ intron enhancer activity, which in turn facilitated the expression upregulation of Igκ via NF-κB and AP-1 signaling pathways in NPC cells [55]. The regulatory mechanisms of tumor-derived Igκ expression are illustrated in Fig. 2B.

### Biological functions and molecular mechanisms of tumor-derived Igs

**Fig. 3:**
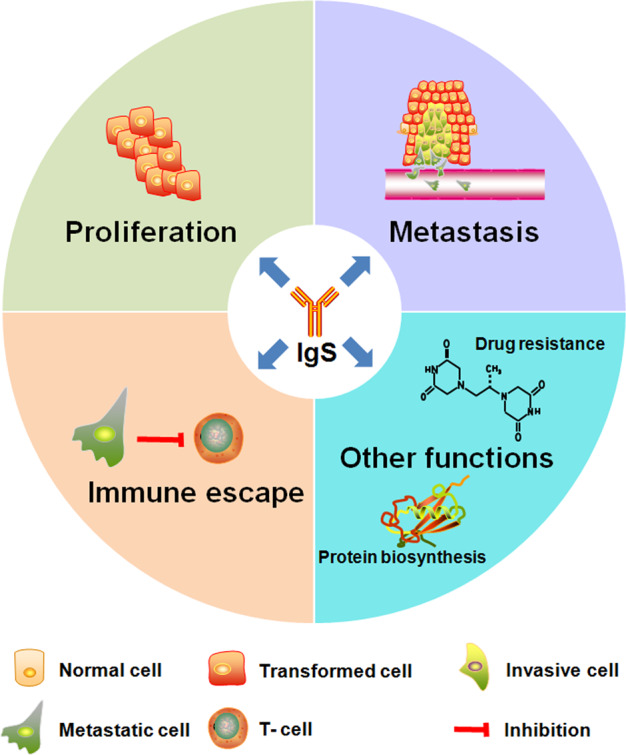
Schematic illustration of biological functions of tumor-derived Igs. Tumor-derived Igs are involved in the following four aspects of tumor development and progression: (1) tumor cell growth and proliferation; (2) tumor cell migration, invasion, and metastasis; (3) tumor immune escape; (4) other biological functions. Other biological functions of tumor-derived Igs are as follows: (1) immunity regulation; (2) promotion of drug resistance; (3) involvement of cancer-associated diabetes; (4) influence of tumor-associated thrombosis; (5) mediation of CSC potential; (6) regulation of cell morphogenesis, cell cycle process, fatty acid biosynthetic process, protein biosynthesis, and antimicrobial (virus, bacterium, and fungus).

According to a multitude of researches, the biological functions of tumor-derived Igs mainly involve four aspects: (1) tumor proliferation; (2) tumor metastasis; (3) tumor immune escape; (4) other functions. The above biological functions of tumor-derived Igs are summarized in Fig. 3.

### Promotion of tumor cell growth and proliferation

In 2003, Qiu et al. found that inhibition of tumor-derived IgG expression by antisense oligodeoxynucleotide and treatment of anti-human IgG antibody induced apoptosis and tumor cell growth suppression in vitro and in vivo. They concluded that tumor-derived IgG, as a growth factor for epithelial cancers, was a new therapy target of human tumors [16]. Reduction of Ig expression led to cell growth inhibition and apoptosis in HT-29, suggesting that tumor-derived Ig strengthened the survival and growth of colon cancer cells [28]. Blockade of tumor-derived Igα inhibited the growth, viability, and access of S phase of cancer cells including HeLa and CNE1 by anti-human Igα antibody, suggesting that tumor-derived Igα promoted cancer cell growth [74]. Chen et al. analyzed the relation between IgG expression and proliferation markers (proliferating cell nuclear antigen, Ki-67, and cyclin D1) in sarcomas tissues based on double immunostaining of the same tissue slide. The results demonstrated that the labeling index of the above proliferation markers was significantly higher in sarcomas tissues with high Igκ expression than that in sarcomas tissues with low Igκ expression, suggesting that IgG may be a useful marker for cell proliferation in sarcomas due to the well correlation between IgG and proliferation markers and tumor grades [33]. Blockade of tumor-derived IgG decreased the viabilities of AML cells (HL-60 and OCI-AML3) and prostate cancer cells (PC3 and Du145) and induced apoptosis using anti-human IgG antibody [35, 38]. Treatment with mouse anti-human IgGγ antibody or silencing of IgG inhibited cell proliferation and/or induced apoptosis of cancer cells (colorectal cancer cell lines LOVO and SW480, cervical cancer cell line HeLa, gastric cancer cell line MGC, and breast cancer cell line MCF-7), which suggested that IgG promoted the above cancer cell proliferation [17, 47, 75]. In 2013, Liang et al. reported that blockade of tumor-derived IgG by either anti-human IgG antibody or antisense oligonucleotides increased cell apoptosis and upregulated cleaved caspase-3 and cleaved poly (ADP-ribose) polymerase in bladder cancer cell line T24 in vitro and in vivo. These findings revealed tumor-derived IgG promoted bladder cancer cell proliferation through inhibiting apoptosis [40].

**Fig. 4:**
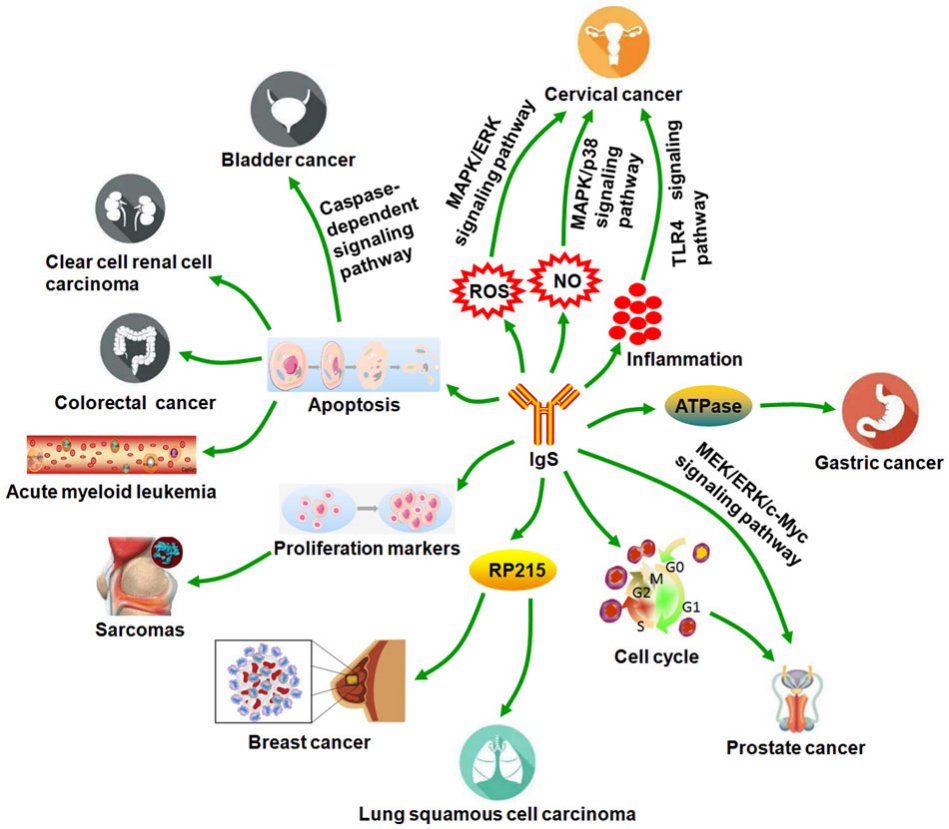
Schematic illustration of the mechanisms that tumor-derived Igs promoted tumor cell growth and proliferation. Tumor-derived Igs augmented multiple tumor cell growth and proliferation through positively or negatively regulating the following cell signaling pathways or biological processes or important molecules, including MAPK/ERK signaling pathway, MAPK/p38 signaling pathway, TLR4 signaling pathway, MEK/ERK/c-Myc signaling pathway, caspase-dependent signaling pathway, apoptosis, cell cycle, ATPase, and proliferation markers. Tumor-derived IgG specifically recognized by RP215 promoted cancer (breast cancer and LSCC) cell proliferation.

In the past few years, our research group got some progress on the biological functions of tumor-derived IgG. Our research group gained 27 potential IgGγ heavy-chain-interacting proteins by co-immunoprecipitation combined with liquid chromatography-tandem mass spectrometry (LC-MS/MS) identification in HeLa cells. We found that at least half of these proteins were closely related to the growth and proliferation of cancer cells. Further studies showed that tumor-derived IgG can strengthen tumor growth and proliferation by inducing the production of low levels of reactive oxygen species [15]. We further found that tumor-derived IgG positively regulated LPS-induced proinflammatory cytokine production through binding to toll like receptor4 (TLR4) and increasing its expression. TLR4 has been reported to facilitate the development of many inflammation-induced cancers such as cervical cancer. These findings strongly implied that IgG may enhance cervical cancer cell proliferation via enhancing TLR4 signaling. For above reasons, we concluded that tumor-derived IgG may be a new therapeutic target in the treatment of inflammation-mediated cancers [76]. To explore the effects of IgG on gastric cancer proliferation and the roles of ATPases in the above phenomenon, we reduced IgG expression in SGC-7901 and HGC-27 cells using siRNA interference. The result showed that downregulation of IgG inhibited gastric cancer cell proliferation by attenuating the activities of total ATPases and three P-type ATPases. Further studies revealed that reduction of IgG increased levels of nitric oxide (NO) by enhancing the activities of all three isoforms of nitrogen oxide synthase. Meanwhile, sodium nitroferricyanide (III) dihydrate, a NO donor, restained cervical cancer cell proliferation via activating the mitogen-activated protein kinase/p38 (MAPK/p38) signaling pathway, while hemoglobin, a NO scavenger, restored the above biological phenomenon. Taken together, our results indicated that tumor-derived IgG augmented cervical cancer cell proliferation via impairing the production of NO. Other studies demonstrated that tumor-derived IgG, which can be recognized by RP215, was mostly located in lamellipodia-like structures of the cell surface. Breast cancer cells MDA-MB-231 with IgG high expression showed a greater ability of proliferation, compared with those with IgG low expression [77]. RP215 was found to induce apoptosis of cancer cells in vitro and inhibited the growth of implanted tumors in nude mouse animal models, suggesting that tumor-derived IgG may facilitate cell growth in many cancer cells, including ovary cancer, lung cancer, breast cancer, and chronic myeloid leukemia [78–82]. Downregulation of IgG expression by siRNA or blockade of IgG with anti-human IgG antibody suppressed cell growth and induced apoptosis in many cancer cells, including pancreas cancer cell lines (BXPC-3 and PANC-1) [18] and ccRCC cell lines (786-0 and ACHN) [41]. For these reasons, researchers concluded that tumor-derived IgG may promote cell growth by inhibiting apoptosis in cancer cells. Silencing IGHG1 expression by siRNA attenuated the colony formation, survival, and cell cycle progression in prostate cancer cells (LNCaP, DU145, and PC3). In addition, silencing IGHG1 expression, which decreased PCNA expression, increased caspase-3 expression, and inactivated MEK/ERK/c-Myc pathway in the above prostate cancer cells. The above data indicated that tumor-derived IGHG1 conferred advantages for the growth and proliferation in prostate cancer cells [83, 84]. Knockdown of tumor-derived IgG by siRNAs inhibited cancer cell proliferation such as SACC cells (SACC-83) [44] and PDAC cells (BxPC-3 and T3M4 cells) [51]. Tumor-derived IgG, which was recognized by RP215, promoted cell proliferation of lung squamous cell carcinoma (LSCC) cell NCI-H520. In addition, RP215 specifically impaired the pro-oncogenic activity of tumor-derived IgG and inhibited in vivo growth of patient-derived xenograft tumors [45]. The mechanisms of action that tumor-derived Igs promote tumor cell growth and proliferation are summarized in Fig. 4.

### Enhancement of tumor cell migration, invasion, and metastasis

Multiple reports showed that tumor-derived Igs enhanced various tumor cell migration, invasion, and metastasis. Silencing of Igκ reduced cell migration ability in AML cell lines including HL-60 and NB4, whereas Igκ overexpression augmented their motility, suggesting that Igκ may play an important role in leukemogenesis and serve as a novel therapeutic target or treating AML [60]. Breast cancer cells with IgG high expression had a higher ability of migration, invasion, and metastasis, compared to those with IgG low expression in vitro and in vivo [77]. Ma et al. tested the expressions of IgG in paraffin-embedded tissue blocks of 68 breast cancers including 40 primary cancers without metastasis and 28 cancers with axillary lymph node metastases. They found that IgG-expressing breast cancer cells were more evenly distributed in the metastatic cancer cells than that in the primary lesion, suggesting that IgG could be a diagnostic biomarker for breast cancer metastasis [12]. Liu et al. found that the ability of migration and invasion in RP215-positive lung adenocarcinoma cells was higher than that in RP215-negative cells. RP215 immunostaining score was negatively correlated with patient prognosis. Downregulation of IgG impaired lung adenocarcinoma cell migration and invasion. For above reasons, they concluded that RP215 can act as a biomarker for the prognosis of lung adenocarcinoma [85]. Sheng at al. demonstrated that silencing of tumor-derived IgG impaired migration and invasion of bladder cancer cells, such as 5637, BIU-87, and EJ [42]. They later confirmed that the migration and invasion abilities of ccRCC, including 786-0 and ACHN cells, were reduced after IgG expression was knocked down by siRNA. High IgG expression correlated well with the poor differentiation and advanced stage of ccRCC [41]. Lv et al. found that downregulation of tumor-derived IgG decreased cell migration and increased expression levels of E-cadherin and alpha-smooth muscle actin in SACC-83 cells. These results indicated that tumor-derived IgG facilitated SACC cell migration through inducing epithelial–mesenchymal transition (EMT) [43, 44].

**Fig. 5:**
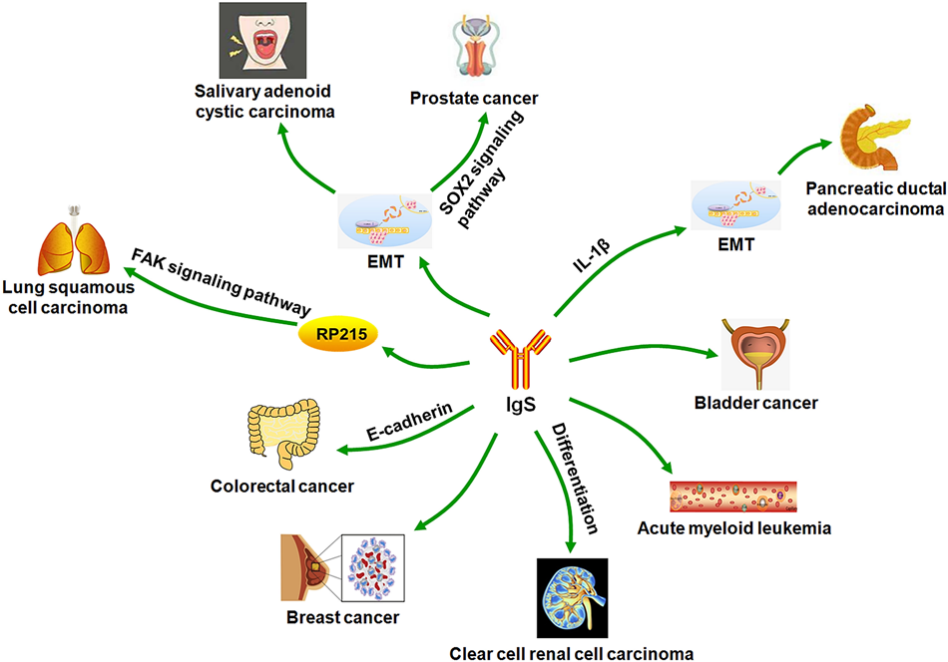
Schematic illustration of the mechanisms that tumor-derived Igs enhanced tumor cell migration, invasion, and metastasis. Tumor-derived Igs augmented multiple tumor cell migration, invasion, and metastasis through positively or negatively regulating the following cell signaling pathways or biological processes or important molecules, including SOX2 signaling pathway, FAK signaling pathway, EMT, differentiation, IL-1β, and E-cadherin.

Other report demonstrated that RP215 recognized tumor-derived IgG interacted with integrin ɑ6β4 and augmented the migration and invasion of LSCC cell NCI-H520 by activating FAK signaling, while RP215 reversed the above biological phenomenon. These findings indicated that tumor-derived IgG could be an attractive target for antibody-based therapy of LSCC [45]. Debris from necrotic PDAC cells induced IL-1β production by M2 macrophages through the TLR4/TRIF/NF-κB signaling pathway, which was further enhanced by tumor-derived IgG from PDAC cells including PANC-1 and BxPC-3. In turn, increased IL-1β promoted metastasis of PDAC cells by inducing EMT [49]. Jiang et al. reported that downregulation of IgG expression impeded the migration and invasion ability of colorectal cancer cells SW480. RNA-seq was employed to profile the transcriptomic changes after silencing of tumor-derived IgG in SW480 cells. Bioinformatics analysis showed that 268 differentially expressed genes (DEGs) were identified, including 71 downregulated genes and 197 upregulated genes. These DEGs were mainly apical junction and EMT related to genes. The top-enriched KEGG pathways of these DEGs were cell adhesion, extracellular matrix receptor interaction, and focal adhesion. They further confirmed that tumor-derived IgG facilitated colorectal cancer invasion and metastasis via binding to E-cadherin [47]. Wang et al. found that IgG expression level was positively correlated with clinical stage, T stage, and metastasis in NSCLC. The gene set enrichment analysis showed that IGHG1 was involved in the following signaling pathways, including cell adhesion, cytokine interaction, and chemokine. On the basis of the above results, they supposed that IgG may act as a poor prognosis factor in NSCLC and participated in cancer invasion and metastasis [46]. Tumor-derived IgG, whose expression was induced by androgen-deprivation therapy and upregulated by SRY (sex-determining region Y)-box 2 (SOX2) in prostate cancer cells such as C4-2 and DU145, promoted the migration and invasion ability of prostate cancer cells through mitogen-activated protein kinase/extracellular signal-regulated kinase and AKT and EMT. The above results suggested that prostate cancer development was promoted through SOX2/tumor-derived IgG signaling pathway [39]. The mechanisms of action that tumor-derived Igs enhance tumor cell migration, invasion, and metastasis are summarized in Fig. 5.

### Involvement in tumor immune escape

**Fig. 6:**
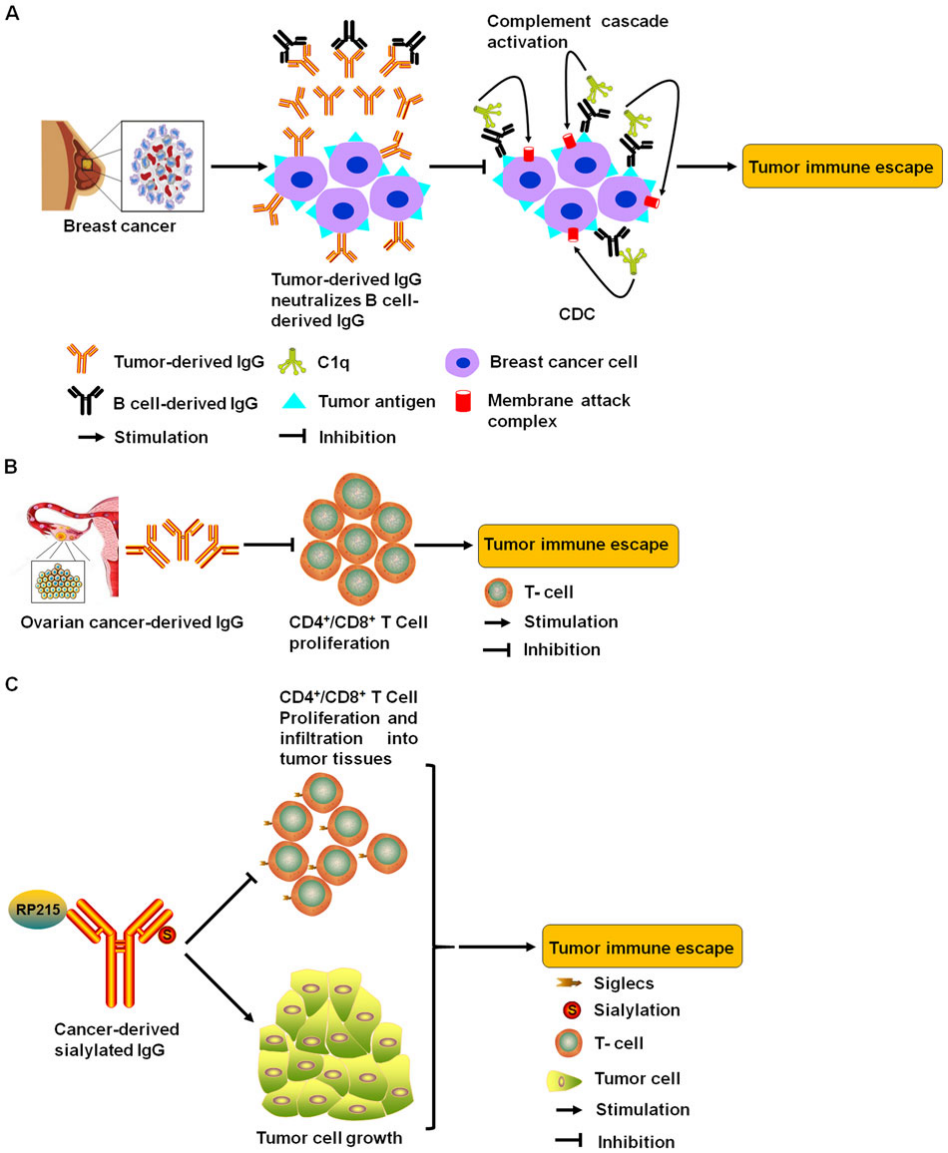
Schematic illustration of the mechanisms that tumor-derived Igs strengthened tumor immune escape. **A** Tumor-derived IgG neutralized B-cell-derived IgG to block CDC in which the formation of a complex, including B-cell-derived IgG, tumor antigen, and C1q, activated complement cascade to lysis tumor cells with the help of membrane attack complex in breast cancer cells. Finally, breast cancer cells escaped from the host immune system. **B** Tumor-derived IgG inhibited the proliferation of CD4^+^ or CD8^+^ T cells from CBMC and CBL to facilitate ovarian cancer immune escape. **C** The sialylation modification mediated the binding between tumor-derived IgG, which was recognized by RP215, and Siglecs on effector CD4^+^ and CD8^+^ T cells, which finally resulted in tumor immune escape due to the immunosuppressive effect of the above T cells and the promotion of tumor cell growth.

There are relatively few reports about Igs involved in the immune escape, compared to those about Igs-mediated tumor cell proliferation and metastasis. Research suggested that IgG and C1q complement was collocated in both primary and metastatic breast cancer lesions, implying that immune complexes may be formed in situ. For the above results, researchers speculated that the above immune complexes may contribute to immune escape of breast cancer cells because tumor-derived IgG, which stops complement-dependent cytotoxicity (CDC) by neutralizing B-cell-derived IgG, keeps the host immune system from attacking tumor cells and promotes tumor growth [12] (Fig. 6A). Liu et al. explored the effect of tumor-derived IgG on T-cell activity. They found that tumor-derived IgG, which was purified from ovarian cancer tissue, could suppress the proliferation of CD4^+^ or CD8^+^ T cells derived from cord blood monocyte cells and cord blood lymphocyte (CBL). In CBL, CD4^+^ or CD8^+^ T cells treated with phosphate buffer saline were more active than those treated with tumor-derived IgG or intravenous Ig. These findings indicated that tumor-derived IgG had the function of immunomodulation. For the above reasons, they concluded that tumor-derived IgG could promote tumor immune escape by inhibiting T-cell proliferation [86] (Fig. 6B).

A new study demonstrated that tumor-derived IgG, which is recognized by RP215, suppressed effector T-cell proliferation and promoted tumor growth by reducing CD4^+^ and CD8^+^ T-cell infiltration into tumor tissues. Further studies showed that tumor-derived IgG directly interacted with sialic acid-binding Ig-type lectins (Siglecs), including Siglec-7 and Siglec-10, on effector CD4^+^ and CD8^+^ T cells by its sialylation of the CH1 domain, which finally led to the immunosuppressive effect of T cells and the promotion of tumor growth. Based on these above findings, they supposed that sialylated tumor-derived IgG may be a new ligand for Siglecs which may serve as potential checkpoint molecules and mediate tumor immune escape [87] (Fig. 6C).

### Other biological functions

The other biological functions of tumor-derived Igs are as follows: (1) tumor-derived Igs regulate immunity [75]; (2) tumor-derived Igs facilitate drug resistance in tumor cells [77]; (3) tumor-derived Igs might be involved in pancreas cancer-associated diabetes [18]; (4) tumor-derived Igs affect tumor-associated thrombosis [88]; (5) tumor-derived Igs mediate cancer stem cell (CSC) potential [77]. Using co-immunoprecipitation combined with LC-MS/MS, our research group identified six potential tumor-derived IgG whole molecule-interacting proteins, including myosin, heavy chain 9, complement component 4A, complement component 4B, acyl-CoA synthetase long-chain family member 3, ribosomal protein L19 and dermcidin, for obtaining clues to study IgG biological functions. Bioinformatic analysis of the above proteins demonstrated that tumor-derived IgG may be involved in the following biological processes: (1) cell morphogenesis; (2) cell migration; (3) cell cycle process; (4) Ig mediated immune response; (5) fatty acid biosynthetic process; (6) protein biosynthesis; and (7) antimicrobial (virus, bacterium, and fungus) [89].

### Similarities and differences between tumor-derived Igs and B-cell-derived Igs

Accumulated evidence demonstrated that there are some similarities and differences between B-cell-derived Igs and tumor-derived Igs. The similarities of the above two types of Igs are as follows: (1) molecular structure. Take IgG, for example, they have identical molecular structure, Y-shaped molecule with twofold symmetry [16]; (2) natural antibody activity. Research has proved that tumor-derived IgM recognized many self-antigens (HEp2 cell antigens) and non-self-antigens (single-stranded DNA, double-stranded DNA, and lipopolysaccharide). TLR9 agonists (CpG 2006, CpG 2078, and GpC) stimulated HeLa MR cells to secrete IgM through the TLR9-MyD88 signaling pathway. These results demonstrated that cancer-derived IgM contained natural antibody activity like B-cell-derived IgM [32]. Their differences are as follows: (1) IgG gene diversity. B-cell-derived IgG gene contains unlimited diversity, while tumor-derived IgG gene displays limited diversity due to its specific restricted patterns of V_H_DJ_H_ recombination and distinct somatic hypermutation mechanism of functional V_H_ region gene [90–93]; (2) glycosylation patterns. B-cell-derived IgG has no O-linked glycans, and has only one N-glycosylation at N297 position of IgG heavy chains and terminal N-acetylneuraminic acid (NeuAc), while tumor-derived IgG has not only O-linked and N-linked glycans but also terminal NeuAc and N-glycolylneuraminic acid (NeuGc) [94–98]; (3) expression regulatory mechanisms. The transcription factors of B-cell-derived IgG include Oct-1, 2, and others, while the transcription factors of tumor-derived IgG include Oct-1 but not Oct-2 [72, 76]; (4) immunoactivity or biological activity. The immunoactivity of tumor-derived IgG is significantly lower than that of B-cell-derived IgG [59] because the former has aberrant glycosylations [99]. (5) biological functions. The functions of B-cell-derived Igs include the following: IgG enhances phagocytosis, neutralizes toxins or viruses, and protects fetus or newborn; IgA protects the mucous membranes against microbial pathogens and the newborns against infection during the first month of life; IgM protects against invasion of blood by microbial pathogens; IgD initiates immune responses; IgE plays a vital role in host defenses against certain parasites and hypersensitivity reaction [7–9]. Unlike B-cell-derived Igs, tumor-derived Igs mainly participate in tumor proliferation, metastasis, immune escape, and other biological behaviors of tumors [15, 16, 100]. It should be pointed out that expression levels of tumor-derived Igs in some tumor cells are higher than that of B-cell-derived Igs, while there is an opposite result in other tumor cells according to this study [14, 15]. Furthermore, there are different Ig expression levels in different tumor cells [14–16]. The similarities and differences between tumor-derived Igs and B-cell-derived Igs are summarized in Table 3.

**Table 3:** The similarities and differences between tumor-derived Igs and B-cell-derived Igs.

	Characteristics	B lymphocyte-derived Igs	Tumor-derived Igs	References
Similarities	Molecular structure	+	+	[16]
	Natural antibody activity	+	+	[35]
Differences	IgG gene diversity	Unlimited diversity	Limited diversity	[90–93]
	Glycosylation patterns	One N-glycosylation at N297 position and terminal NeuAc	O-linked and N-linked glycans, terminal NeuAc and NeuGc	[94–98]
	Expression regulatory mechanisms	Transcription factors include Oct-1, Oct-2, NF-κB, B-cell Oct-binding factor-1 and others	Transcription factors include Oct-1 but not Oct-2	[72, 76]
	Immunoactivity	Normal	Low	[59, 99]
	Biological functions	Phagocytosis and neutralize toxins (IgG); mucosal immunity and neonatal immunity (IgA); protection against invasion of blood by microbial pathogens. (IgM); initiation of immune responses (IgD). Antiparasitic immunity and antihypersensitivity (IgE)	Tumor proliferation, metastasis, immune escape, and other biological behavior of tumors	[7–9, 15, 16, 100].

## Conclusions

Although outstanding progress has been achieved in the research about tumor-derived Igs in the past decades, more unknown fields of tumor-derived Igs still need to be explored. Future research can be carried out from the following aspects: (1) structural characteristics of tumor-derived Igs. (2) physicochemical properties of tumor-derived Igs such as biological activities. (3) mechanisms for rearrangement and expression regulation of tumor-derived Igs. (4) exact mechanisms of Igs-mediated cell behaviors, including proliferation, metastasis, immune escape, etc. These mechanisms can be explored from the point of view of related cellular signaling pathways such as MAPK signaling pathways, TLRs signaling pathways, mitochondria-caspase signaling pathway, EMT, etc.

In addition to the above future research fields, the clinical applications of tumor-derived Ig based on the above functions and mechanisms should also be explored. For example, tumor-derived IgG can act as a new therapeutic target for many tumors in the future such as salivary adenoid cystic carcinoma [44], colorectal cancer [17, 48], sarcoma [33], and parathyroid cancer [50]. Tumor-derived IgG and IgM might serve as a potential target for AML therapy and useful markers for monitoring minimal residual AML [35, 101]. Tumor cell-derived IgG may be a target to prevent and treat tumor-associated thrombosis because it interacted directly with FcγRIIa and activated platelets [88]. In addition, monoclonal antibodies targeting tumor-derived IgG as neutralizing antibodies can also be used as antitumor drugs. To our excitement, RP215 monoclonal antibody, which recognized unique carbohydrate-associated epitope(s) in cancer cells, can be applied in immunodiagnostics and developed to antibody-based anticancer drugs [31]. Peptide mimics (R2 and R42) of cancer-associated antigen 215C, which is a carbohydrate-associated epitope of an Ig molecule expressed by cancer cells, had been identified to serve as a tumor vaccine candidate [102]. In summary, tumor-derived Igs play a pivotal role in carcinogenesis. More clinical applications of tumor-derived Igs still need to be unveiled in the future.
